# Examining the moderating effects of biopsychosocial factors on the relationship between HIV-related depression and cognitive function among adolescents living with HIV in Tanzania: A protocol for an analytical cross-sectional study

**DOI:** 10.1371/journal.pone.0313223

**Published:** 2025-01-06

**Authors:** Siku Kitoki, Saada Ali Seif, Golden Mwakibo Masika

**Affiliations:** 1 Department of Clinical Nursing, School of Nursing and Public Health, University of Dodoma, Dodoma, Tanzania; 2 Department of Public Health and Community Nursing, School of Nursing and Public Health, University of Dodoma, Dodoma, Tanzania; Elizabeth Glaser Pediatric AIDS Foundation, UNITED REPUBLIC OF TANZANIA

## Abstract

**Background:**

Adolescents living with HIV face unique challenges, including mental health issues such as depression and cognitive dysfunction. Despite this significant burden, there is a lack of evidence focusing on this population. This study therefore aims to examine the moderating effects of biopsychosocial factors on the bi-directional relationship between HIV-related depression and cognitive function among adolescents living with HIV in the Mbeya region, Tanzania.

**Methods:**

The analytical cross-sectional study will be conducted in public healthcare facilities in the Mbeya region, involving 207 adolescents living with HIV who will be selected using a systematic random sampling technique. Depression will be assessed using a standardized PHQ-9 tool, while cognitive functioning will be evaluated using a validated Montreal Cognitive Assessment Tool Version 8.3. A biopsychosocial model will be used to guide this study. Descriptive statistics will be used to describe the frequency distribution of the study variables, a chi-square test will be used to describe the relationship between the variables and a binary logistic regression model will be used to predict the moderating effects of biopsychosocial factors on the relationship between HIV-related depression and cognitive function. The odds ratio and a 95% confidence interval will be reported, and the p-value of 0.05 will be considered statistical significance. This study is expected to be conducted for three months. The high prevalence of mental health issues among HIV-positive adolescents, the detrimental effect of HIV on cognitive development, and the lack of studies focused on this population raised the need to conduct this study.

**Discussion:**

The findings of this study will inform the development of a comprehensive integrated care model that addresses both the mental and cognitive aspects of living with HIV during adolescence. Moreover, the identification of factors associated with depression and cognitive performance will help healthcare providers in identifying at-risk adolescents and proper management of the conditions. This study’s results will also help reveal areas that need in-depth exploration that will lead to a better understanding of the responsible factors.

## Introduction

Adolescents Living with HIV (ALHIV) face numerous challenges related to their health and well-being, including mental health issues such as depression and cognitive dysfunction. Globally, an estimated 1.01 million adolescents aged 15–19 years were living with HIV in 2023, with sub-Saharan Africa bearing the greatest burden, 89% [1]. In Tanzania, HIV prevalence among adolescents remains high at 5.8% [2], and in the Mbeya region it is 9.6% [3], however, the prevalence of adolescents’ age groups is not specified but stated to be high according to Tanzania’s health reports and studies [4].

The psychosocial impact of HIV on adolescents can be profound, leading to increased vulnerability to mental health disorders like depression. Depression is a serious mental health condition whereby a person experiences persistent depressed mood, loss of interest in pleasurable activities, feelings of guilt or low self-worth, disturbed sleep and appetite, difficulty thinking, and poor concentration, the symptoms must occur for at least two-week period, significantly affecting how a person feels, thinks, and functions in day-to-day life [5, 6]. Different studies have reported a higher prevalence of depression among people living with HIV/AIDS, ranging from 32.2% to 57% in Pakistan, China, and India [7–9]. However, most of these studies involve adult populations. The few studies conducted among children and adolescents living with HIV also showed a relatively higher prevalence ranging from 18.9% to 27% in Malawi, Rwanda, and Tanzania [10–12].

Furthermore, it is well evidenced that people living with HIV tend to develop a spectrum of cognitive, motor, and mood conditions known as HIV-associated neurocognitive disorder (HAND), which induce difficulties with attention, concentration, and memory, loss of motivation, irritability, depression, and slow movements [13]. Data from a meta-analysis study suggests that 42.6% of people living with HIV suffer from HAND [14]. Cognitive impairments in particular can significantly impact daily functioning, academic performance, and overall quality of life among adolescents living with HIV [15]. For instance, a study conducted on children living with HIV showed that HIV-positive children had lower IQ scores in comparison to HIV-negative children [16]. These shreds of evidence suggest that the problem needs significant attention.

Depression and cognitive failure are closely connected. Evidence shows that cognitive impairment is among the most prominent components of major depressive disorder [17], and it is an important complication of depression among people living with HIV [18]. Despite the significant burden of depression and cognitive dysfunction among adolescents living with HIV, there is limited research examining the interaction between HIV serostatus and depression on cognitive dysfunction focusing on this population, particularly in low-resource settings such as Tanzania. Factors associated with depression and cognitive impairments among adults are known, which include anxiety, obesity, older age, low education, being single, physical inactivity, lack of social support and hospitalization and late-life depression [19]. However, not much has been explored on the side of adolescents. Therefore, understanding the factors associated with these conditions is crucial for developing targeted interventions and support programs tailored to the needs of adolescents living with HIV.

Therefore, the overall aim of this study is to examine the moderating effects of biopsychosocial factors on the bi-directional relationship between HIV-related depression and cognitive function, among adolescents living with HIV in the Mbeya region, Tanzania. Specifically, this study will assess the prevalence of depression and its associated factors, the prevalence of cognitive dysfunction and its associated factors, and the association between depression and cognitive dysfunction among adolescents living with HIV in Mbeya Region, Tanzania. This study will be guided by the biopsychosocial model, which recognizes that an individual’s health, behavior, and functioning are influenced by the complex interplay of three main factors, which are biological (physiological, genetic, hormonal, and medical conditions), psychological (cognitive process, emotional states, personality traits, coping strategies, and stress response), and social factors (interpersonal relationships, family dynamics, socioeconomic status, cultural norms and beliefs, environmental influences, and societal structure).

The findings of this study are expected to inform the health care provider to improve the practice of assisting ALHIV and they are expected to be useful among healthcare givers and relevant stakeholders in innovating programs for enhancing services and lives of ALHIV. The study results are expected to potentially contribute to the government’s (Tanzania) efforts to broaden the understanding of the prevalence of HIV-related mental disorders, diversify decision-making, and shape policy-making through expected options from this study.

## Methodology

### Study area

This study will be conducted in the Mbeya Region of Tanzania. The Mbeya region is located in the southern highlands of Tanzania, and it is among the top three areas with a high prevalence of HIV at 9.6% [20]. Participants will be recruited from all four major healthcare facilities that provide HIV care and treatment (CTC) in the region, including Mbeya Zonal Referral Hospital (MZRH), Mbeya Region Referral Hospital (MRRH), Military Hospital Mbeya (MH-MBY), and District Designated Hospital (DHH).

### Study design

An analytical cross-sectional study will be used.

### Study population

Adolescents aged 15–19 years living with HIV who will be receiving care and treatment at selected healthcare facilities will be involved. The choice of older adolescents (15–19 years) compared to younger adolescents is because this is a period in which individuals with HIV face many challenges, like the transition to adult care, and navigating treatment and disclosure adherence. All these contribute to the risk of developing depression and cognitive problems. The inclusion criteria will be adolescents who know their HIV serostatus and who will agree to participate, while the study will exclude those who will be seriously ill and those who are under 18 years old but not accompanied by their adult caregiver.

### Sample size and sampling technique

The sample size is calculated using a formula for a cross-sectional study $n=\frac{Z^{2}P\left(1-P\right)Z^{2}P(1-P)}{d^{2}}$ (Cochran, 1963), where n = the required sample size, Z = 1.96, which corresponds to a significance level of 0.05 and a confidence level of 95%, P = 16%, which is the prevalence of depression among adolescents receiving care and treatment for HIV/AIDS in the Kilimanjaro Region in Tanzania, and d = 0.05, which is the desired level of precision. Therefore, n $=\;\frac{1.96^{2}\;x\;0.16\left(1-0.16\right)1.96^{2}\;x\;0.16(1-0.16)\;}{0.05^{2}}\;$ = 207. Considering the attrition rate of 10%, the required sample size is 232 participants.

### Sampling technique

The study will use a systematic random sampling technique, whereby, the sampling frame will be the total number of adolescents attending CTC on the day of data collection, and the k^th^ interval will be calculated using a formula k^th^ = N/n [21]. where N = sampling frame and n = sample size required on that particular day.

### Data collection method and procedure

Data will be collected using a self-administered paper-based questionnaire. A principal investigator and two trained research assistants who are healthcare providers will collect the data. After completing his/her routine care, the adolescent will be approached and asked for his/her consent to participate or assent for those under 18 years old after getting consent from his/her caregiver accompanying him/her. The participant will be placed in a quiet private space, and clear instructions on how to complete the questionnaire will be provided. They will be encouraged to answer all questions, but they may skip any questions they may not feel comfortable answering, and confidentiality will be highly ensured. Completed questionnaires will be recollected on the same day and will be kept in a secured briefcase.

### Data collection tools

Four data collection tools will be used, the first is PHQ-A- (Patient Health Questionnaire Modified for Adolescents). This is the standard structured tool that will be used to assess depression in adolescents. The tool has 9 items in a Likert scale format, and it was validated and used in Tanzania [22], with a Cronbach alpha of 0.89 [23]. The second tool is MoCA (Montreal Cognitive Assessment) version 8.3. This standard tool is designed to detect mild cognitive impairment. It comprises 30 points for the items classified into eight domains (executive function, naming, memory, attention, language, abstraction, delayed recall, and orientation). MOCA is considered a reasonable screening tool for cognitive assessment among people living with HIV demonstrating an area under the curve of 0.71 of the receiver operating characteristic curve [24]. The Cronbach alpha of this tool measured from other populations in Tanzania is 0.78 [25]. The third tool is a stigma scale; this tool is used to assess a person’s feeling of distress from the reaction of other people to his/her HIV condition. The tool comprises 28 items, however, 19 questions related to discrimination and stigma, bullying, and disclosure were modified and included in the initial questionnaire, which has a Cronbach alpha of 0.87 [26]. The fourth tool is a Multidimensional Scale of Perceived Social Support (MSPSS). This tool assesses social support that a person receives from family, peers, or significant others, which may affect psychologically, socially, and physically [27]. This tool is validated and used in South Africa for youth [28] The tool has 12 items in a Likert scale format, with a Cronbach alpha range from 0.8 to 0.9 [27].

### Variable definitions and measurement

Dependent variables are depression and cognitive function.

### Depression

In this study, depression is defined as the tendency of the adolescent to express persistent feelings of irritable mood, loss of interest in a pleasurable activity, slowed thinking, less attention, poor concentration, sleep disturbance, change in appetite and suicidal thoughts during two weeks period, which affect how teens think, feel, and behave [5, 29]. This will be measured by 9 items in a 4-point Likert scale on the rate of depressive symptoms experienced over the past two weeks. The response will be scored 0 = not at all, 1 = several days, 2 = more than half the days, and 3 = nearly every day. The total score ranges from 0–27, with higher scores indicating more severe symptoms. A total score of 0–4 means no depression, 5–9 is mild depression, 10–14 is moderate depression, 15–19 is moderately severe depression, and 20–27 is severe depression [30].

### Cognitive function

In this study, cognitive function refers to the adolescents’ brain ability to process information through planning, solving problems, concentration and attention, having good memory, visual-spatial ability, and processing speed [15]. This variable will be measured by eight domains of the level of cognitive function. The domains are:

- Executive function: This domain measures cognitive processes like conceptual thinking, planning and problem-solving, and cognitive flexibility. This variable will be assessed by 3 items measured on a binary scale (pass/fail). For example, a participant will be tasked with trail making, copying an object, drawing a clock and locating the time given. A score value will be assigned, 1 for pass and 0 for fail. The total score in this domain is 5 points (2 points for tasks 1 and 2, and 3 points for task 3).
- Naming: This domain measures the semantic memory of an individual. The variable will be assessed by 3 items measured on a binary scale (pass/fail). For example, a participant is asked to name three animals of low familiarity that will be presented to him using images drawn on paper. A score value will be assigned, 1 for pass and 0 for fail. The total score in this domain is 3 points.
- Memory: This domain measures the immediate and delayed recall. The variable will be assessed by 5 items measured on a binary scale (pass/fail). For example, a participant is asked to immediately repeat as many of the words as possible after they are read aloud (for immediate recall) and repeat the same words after 5 minutes (for delayed recall). A score value will be assigned, 1 for pass and 0 for fail. The total score in this domain is 5 points.
- Attention/ concentration: This domain measures various aspects of attention including divided attention, working memory, and sustained attention. The variable will be assessed by 3 items measured on a binary scale (pass/fail) on digit span (ability to repeat the list of digits forward and backwards after reading aloud), vigilance (ability to tap a hand every time a specific letter is presented in a series of letters), and serial 7s (ability to count backwards from 100 by serially subtracting 7). A score value will be assigned, 1 for pass and 0 for fail. The total score in this domain is 6 points (2 points for digit span, 1 point for vigilance, and 3 points for serial 7s).
- Language: This domain measures language comprehension, verbal working memory, and language production. The variable will be assessed by 3 items measured on a binary scale (pass/fail) on whether a participant can repeat two syntactically complex sentences and can retrieve words. A score value will be assigned, 1 for pass and 0 for fail. The total score in this domain is 3 points. Abstraction: This domain measures thinking and reasoning skills. The variable will be assessed by 2 items measured on a binary scale on whether a participant can identify similarities between objects and concepts presented. A score value will be assigned, 1 for pass and 0 for fail. The total score in this domain is 2 points.
- Delayed Memory: This domain measures the ability to recall information after a delay. This variable is assessed by 5 items measured on a binary scale (pass/fail) on whether a participant can recall 5 words previously presented in the memory domain, recall after given cues, or be presented with a recognition task. A score value will be assigned, 1 for pass and 0 for fail. The total score in this domain is 5 points (1 point for each item recalled).
- Orientation: This domain measures awareness of current time and place. The variable will be assessed by 2 items measured on a binary scale (pass/fail) on temporal orientation and spatial orientation. Temporal orientation measures whether a participant can provide the current date, day, month, and year, and spatial orientation measures whether a participant can identify the city and the country he/she is currently in. A score value will be assigned, 1 for pass and 0 for fail. The total score in this domain is 6 points (4 points for temporal orientation and 2 points for spatial orientation).

The total score of all domains is 30 points, a score of 26 and above is regarded as intact cognitive function, and a score below 26 indicates the presence of mild cognitive dysfunction.

### Independent variable

According to the biopsychosocial model, the independent variables are the biological, social and psychological factors. The biological factors in this study are age, sex, substance abuse, adherence to medication, genetics, smoking, and diet, while the psychological factors are personality traits, coping strategies, and stress response patterns, and the social factors are family and peer relationships and support, experiencing bullying, stigma, and discrimination.

### Data analysis plan

Data will be entered in SPSS, a software package version 25, and cleaned before being analyzed. Descriptive statistics will describe the frequency distribution of all study variables and the prevalence of depression and cognitive dysfunction. A chi-square test will be used to describe the relationship between depression, cognitive dysfunction, and background characteristics, and a binary logistic regression model will be used to predict factors associated with HIV-related depression and cognitive dysfunction and to estimate the effect of depression on cognitive dysfunction. Odds ratio and a 95% confidence interval will be reported, and a p-value of 0.05 will be considered statistical significance.

### Duration of the study

The study is expected to be conducted for 3 months in the Mbeya region, Tanzania.

## Discussion

This study will provide insight into the cognitive and mental health challenges experienced by adolescents living with HIV. The findings of this study will inform the development of a comprehensive integrated care model that addresses both the mental and cognitive aspects of living with HIV during adolescence. Moreover, the identification of factors associated with depression and cognitive performance will help healthcare providers in identifying at-risk adolescents and proper management of the conditions. This study’s results will also help reveal areas that need in-depth exploration that will lead to a better understanding of the responsible factors.

## Supporting information

S1 File Structured questionnaire. (DOCX)
