# Supplementary material for: Examining the moderating effects of biopsychosocial factors on the relationship between HIV-related depression and cognitive function among adolescents living with HIV in Tanzania: A protocol for an analytical cross-sectional study
Source: PLoS One. 2025 Jan 6;20(1):e0313223. doi: 10.1371/journal.pone.0313223 (PMC11703066; doi:10.1371/journal.pone.0313223)
Supplement: S1 File — (DOCX) [file pone.0313223.s001.docx]

**“S1 file”: Questionnaires sheet**

**General Instructions**

 No need of writing your name

 In all cases where answer options are available, please tick (√) in the box provided

 For blank spaces provided write the answer over there.

 For scale typed questions please circle your preferred level of agreement

Thank you in advance for your honest cooperation.

Part I: Demographic Information

1. How old are you?..............

2. What is your sex?..............

a) Male

b) Female.

3. What is your place of residence?

a) Urban.

b) Rural.

4. What education level have you achieved

a) primary level

b) secondary level

c) college level

d) Informal education

5. What is your current marital status?

a. I am single

b. I am married

6. What is your current occupation of your parents?

a) Employed

b) Self-Employed

c) Unemployed.

7. Living Arrangement:

a) live alone

b) parents

c) guardians

8. Are you currently taking prescribed ARV medications? No / Yes

9. If yes, what type of ARV medication(s) are you taking?...........

10. Adherence to ARV Medication:

a. On a scale of 1-5, how would you rate your adherence to your prescribed medication regimen? Put a tick to one of the boxes accordingly

1) Poor adherence

2) below average adherence

3) average adherence

4) Above-average adherence

5. Excellence adherence

| 1 | 2 | 3 | 4 | 5 |
| --- | --- | --- | --- | --- |
|  |  |  |  |  |

b. Do you often forget to take your ARV medication? No /Yes

c. How often do you visit your healthcare provider for routine ARV taking

1) after one month

2) after three months

3) after six months

4) after one year

11. Do you consume alcohol? No /Yes

12. Do you smoke? No / Yes

13. If yes, how many cigarettes do you smoke per day?

a) 1-5

b) 6-10

c) 11-20

4) 20+

14. Does anyone in your immediate family (parents, siblings) have a history of mental illness? No /Yes

15. In the past 24hrs did you consume healthy fats and oils like nuts, seed pastes, and avocado? No/yes

16. In the past 24 hours, did you consume a variety of fruits and vegetables like spinach, chines, carrots, salad vegetables and fruits like oranges, mangoes, apples, pears etc Yes/ No

17. Do you consume starchy foods every day? like slices of bread, rice, pasta, ugali etc No/Yes

18. Do you consume protein-rich foods like meat, fish, eggs, poultry, legumes, and seeds daily? No/ Yes

19. In the past 24hrs did you consume Dairy products like milk, cheese, and yoghurt daily? No/Yes

20. Do you rely on government assistance progress or social services to meet your family's needs

1) Yes

2) Yes

21 Have you encountered barriers to accessing medical treatment due to financial limitations

1) Yes

2) Yes

22. Do you have access to reliable transport for work, school, or essential activities?

1) Yes

2) No

23. Have you experienced homelessness or housing instability in the past?

1) Yes

2) No

24. Do you have reliable transportation to reach the health care facility?

1) Yes

2) No

25. How far do you have to travel to reach the nearest healthcare facility

1) Within 15 minutes

2) Within 15 to 30 minutes

3) Within 30 to 60 minutes

4) More than 60 minutes

26. Do you currently have health insurance coverage?

1) Yes

2) No

27. Have you ever delayed seeking medical treatment due to concern about cost?

1) Yes

2) No

28. Do you have a regular primary care physician or health care provider?

1) Yes

2) No

Part II: PHQ-a for screening depression

Introduction: How often have you been bothered by each of the following symptoms during the past two weeks. For each symptom put an X in the box beneath the answer that best describe how you have been feeling.0=Not at all, 1=Several days, 2=More than half the days, 3=Nearly every day.

|  | | | | |
| --- | --- | --- | --- | --- |
|  | (0) | (1) | (2) | (3) |
| 1. Feeling down, depressed, irritable, or hopeless? |  |  |  |  |
| 2. Little interest or pleasure in doing things? |  |  |  |  |
| 3. Trouble falling asleep, staying asleep, or sleeping too much? |  |  |  |  |
| 4. Poor appetite, weight loss, or overeating. |  |  |  |  |
| 5. Feeling tired, or having little energy? |  |  |  |  |
| 6. Feeling bad about yourself – or feeling that you are a failure, or that you have let yourself or your family down? |  |  |  |  |
| 7. Trouble concentrating on things like school work, reading, or watching TV? |  |  |  |  |
| 8. Moving or speaking so slowly that other people could have noticed?  Or the opposite – being so fidgety or restless that you we’re moving around a lot more than usual? |  |  |  |  |
| 9. Thoughts that you would be better off dead, or of hurting yourself in some way? |  |  |  |  |

| **VISUOSPATIAL / EXECUTIVE** | | | **Copy bed** | | | | | | | | **Draw CLOCK (Five past ten)**  (3 points)  **[ ] [ ] [ ]**  **Contour Numbers Hands** | | | | | | | | | | **POINTS** |
| --- | --- | --- | --- | --- | --- | --- | --- | --- | --- | --- | --- | --- | --- | --- | --- | --- | --- | --- | --- | --- | --- |
| **VISUOSPATIAL / EXECUTIVE**  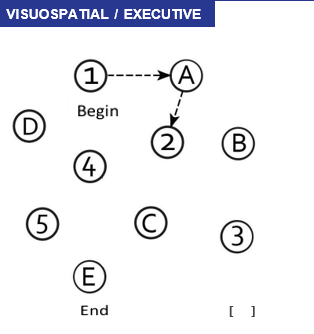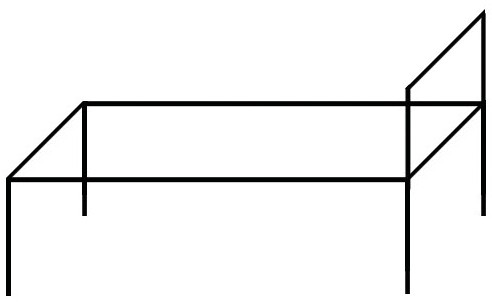  **[ ] [ ]**  **[ ] [ ]** | | | | | | | | | | |  |  |  |  |  |  |  |  |  |  |  |
|  |  |  |  |  |  |  |  |  |  |  |  |  |  |  |  |  |  |  |  |  | **/ 5** |
| **NAMING** |  | | | | | | | | | | | | | | | | | | | | **/ 3** |
| 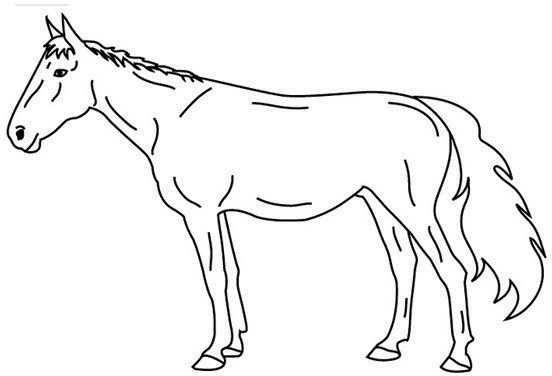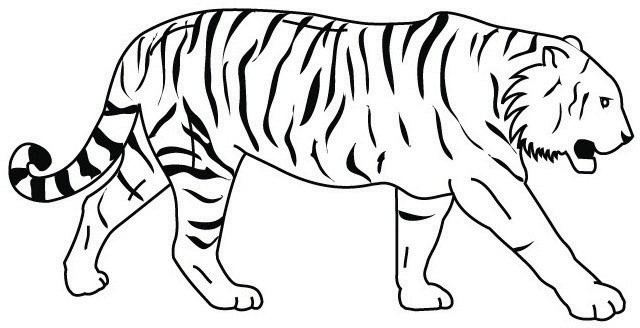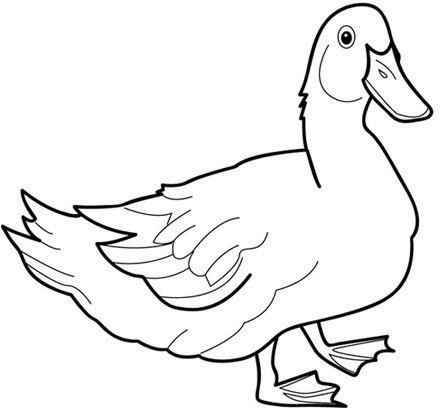  **[ ] [ ] [ ]** | | | | | | | | | | | | | | | | | |  |  |  |  |
| **MEMORY** | Read list of words, subject must | | | |  | | LEG | | COTTON | | | SCHOOL | | | | TOMATO | WHITE | | | | **NO POINTS** |
| repeat them. Do 2 trials, even if 1st trial is successful. Do a recall after 5 minutes. | | | | | 1st TRIAL | |  | |  | | |  | | | |  |  | | | |  |
|  |  |  |  |  | 2nd TRIAL | |  | |  | | |  | | | |  |  | | | |  |
| **ATTENTION** | Read list of digits (1 digit / sec.). Subject has to repeat them in the forward order. **[ ]** 2 4 8 1 5 | | | | | | | | | | | | | | | | | | | | **/ 2** |
| Subject has to repeat them in the backward order. **[ ]** 4 2 7 | | | | | | | | | | | | | | | | | | | | |  |
| Read list of letters. The subject must tap with his hand at each letter A. No points if ≥ 2 errors.  **[ ] F B A C M N A A J K L B A F A K D E A A A J A M O F A A B** | | | | | | | | | | | | | | | | | | | | | **/ 1** |
| Serial 7 subtraction starting at 60. **[ ]** 53 [ ] 46 **[ ]** 39 **[ ]** 32 **[ ]** 25  4 or 5 correct subtractions: **3 pts,** 2 or 3 correct: **2 pts,** 1 correct: **1 pt,** 0 correct: **0 pt** | | | | | | | | | | | | | | | | | | | | | **/ 3** |
| **LANGUAGE** | Repeat: The child walked his dog in the park after midnight. **[ ]** | | | | | | | | | | | | | | | | | | | | **/ 2** |
| The artist finished his painting at the right moment for the exhibition. **[ ]** | | | | | | | | | | | | | | | | | | | | |  |
| Language Fluency. Name maximum number of words in one minute that begin with the letter B. **[ ]**  (N ≥ 11 words) | | | | | | | | | | | | | | | | | | | | | **/ 1** |
| **ABSTRACTION** | Similarity between e.g. orange - banana = fruit **[ ]** hammer - screwdriver **[ ]** matches - lamp | | | | | | | | | | | | | | | | | | | | **/ 2** |
|  | | | | | | | | | | | | | | | | | | | | |  |
| **DELAYED RECALL** | (MIS) | Has to recall words WITH NO CUE | | LEG  **[ ]** | | COTTON  **[ ]** | | SCHOOL  **[ ]** | | TOMATO  **[ ]** | | | WHITE  **[ ]** | | Points for UNCUED  recall only | | | | | | **/ 5** |
| Memory Index Score (MIS) | X3 |  |  |  |  |  |  |  |  |  |  |  |  |  |  |  |  |  |  |  |  |
|  | X2 | Category cue | |  | |  | |  | |  | | |  | | MIS = / 15 | | | | | |  |
|  | X1 | Multiple choice cue | |  | |  | |  | |  | | |  | |  |  |  |  |  |  |  |
| **ORIENTATION** | **[ ]** Date **[ ]** Month **[ ]** Year **[ ]** Day **[ ]** Place **[ ]** City | | | | | | | | | | | | | | | | | | | | **/ 6** |
|  | | | | | | | | | | | | | | **TOTAL** | | | |  |  | **/ 30** | |

Part III: Cognitive functions assessment tools

**Part IV:** These questions answer HIV-related stigma, discrimination, disclosure, and bullying.

Find below a list of sentences, for each one of them, you need to check off the answer that best suits you by put the letter X in the square which you feel is the best answer to you.

1. Strongly agree
2. Agree
3. Neither agree nor disagree
4. Disagree
5. Strongly disagree

| **Answer all the questions without exception. Don’t spend too much time thinking about the answer, as your first impression is important. Statement** | **Response**  **1 2 3 4 5** | | | | | |
| --- | --- | --- | --- | --- | --- | --- |
| 1. People have avoided you because of being infected with HIV? |  |  |  |  |  |  |
| 2. Sometimes you feel that you are being talked down because of being HIV infected? |  |  |  |  |  |  |
| 3. Do you think some people with HIV infection are dangerous |  |  |  |  |  |  |
| 4. Always, do you feel lonely because of being HIV infected? |  |  |  |  |  |  |
| 5. You are angry with the way people have reacted to your HIV status? |  |  |  |  |  |  |
| 6.Have you been discriminated against by employers because of being HIV infected? |  |  |  |  |  |  |
| 7.Have you been discriminated against in education because of your HIV status? |  |  |  |  |  |  |
| 8. Have you been discriminated against by health professionals because of infected with HIV? |  |  |  |  |  |  |
| 9. Have you been discriminated against by the community because of being infected with HIV? |  |  |  |  |  |  |
| 10.Have you been experienced form of discrimination from your friends because of HIV infection? |  |  |  |  |  |  |
| 11. Are you scared of how other people will react if they find out about your HIV status? |  |  |  |  |  |  |
| 12.Are you careful to whom to say that you have HIV infection? |  |  |  |  |  |  |
| 13. Are you worry about telling people you receive antiretroviral drugs? |  |  |  |  |  |  |
| 14. Do you work hard to keep your HIV status secret? |  |  |  |  |  |  |
| 15. Do you feel the need to hide your HIV status from your friends? |  |  |  |  |  |  |
| 16. Have you been bullied by your fellow students because of being infected with HIV? |  |  |  |  |  |  |
| 17. Having HIV infected makes you feel that life is unfair? |  |  |  |  |  |  |
| 18. People have insulted you because of being HIV infected? |  |  |  |  |  |  |
| 19. Have you been harassed because of your HIV infection? |  |  |  |  |  |  |

**PART FIVE**: Multidimensional scale of perceived social support (MSPSS)

**Instructions**: We are interested in how you feel about the following statements. Read each statement carefully and indicate how you feel about each statement.

Circle the “1” if you Very Strongly Disagree

Circle the “2” if you Strongly Disagree

Circle the “3” if you Mildly Disagree

Circle the “4” if you are Neutral

Circle the “5” if you Mildly Agree

Circle the “6” if you Strongly Agree

Circle the “7” if you Very Strongly Agree

Put the letter X for what you feel is the best answer below the number provided below

1. There is a special person who

is around when you’re in need? 1 2 3 4 5 6 7

2. There is a special person with

whom you can share joys and sorrows? 1 2 3 4 5 6 7

3. Do your family really tries to help you? 1 2 3 4 5 6 7

4. Do you get the emotional help & support

you need from my family? 1 2 3 4 5 6 7

5. Do you have a special person who is

a real source of your comfort? 1 2 3 4 5 6 7

6. Do your friends really try to help you? 1 2 3 4 5 6 7

7. Do you count on your friends when

things go wrong? 1 2 3 4 5 6 7

8. Can you talk about your problems with

your family? 1 2 3 4 5 6 7

9. Do you have friends with whom you can

share your joys and sorrows? 1 2 3 4 5 6 7

10. There is a special person in your

life who cares about your feelings? 1 2 3 4 5 6 7

11. Does your family is willing to help you

make decisions? 1 2 3 4 5 6 7

12. Can you talk about your problems with

your friends? 1 2 3 4 5 6 7
